# Extended Bidomain Modeling of Defibrillation: Quantifying Virtual Electrode Strengths in Fibrotic Myocardium

**DOI:** 10.3389/fphys.2019.00337

**Published:** 2019-04-03

**Authors:** Jean Bragard, Aparna C. Sankarankutty, Frank B. Sachse

**Affiliations:** ^1^Department of Physics and Applied Mathematics, University of Navarra, Pamplona, Spain; ^2^Nora Eccles Harrison Cardiovascular Research and Training Institute, University of Utah, Salt Lake City, UT, United States; ^3^Department of Biomedical Engineering, University of Utah, Salt Lake City, UT, United States

**Keywords:** defibrillation, cardiac tissue, fibrosis, computational modeling, multidomain modeling

## Abstract

Defibrillation is a well-established therapy for atrial and ventricular arrhythmia. Here, we shed light on defibrillation in the fibrotic heart. Using the extended bidomain model of electrical conduction in cardiac tissue, we assessed the influence of fibrosis on the strength of virtual electrodes caused by extracellular electrical current. We created one-dimensional models of rabbit ventricular tissue with a central patch of fibrosis. The fibrosis was incorporated by altering volume fractions for extracellular, myocyte and fibroblast domains. In our prior work, we calculated these volume fractions from microscopic images at the infarct border zone of rabbit hearts. An average and a large degree of fibrosis were modeled. We simulated defibrillation by application of an extracellular current for a short duration (5 ms). We explored the effects of myocyte-fibroblast coupling, intra-fibroblast conductivity and patch length on the strength of the virtual electrodes present at the borders of the normal and fibrotic tissue. We discriminated between effects on myocyte and fibroblast membranes at both borders of the patch. Similarly, we studied defibrillation in two-dimensional models of fibrotic tissue. Square and disk-like patches of fibrotic tissue were embedded in control tissue. We quantified the influence of the geometry and fibrosis composition on virtual electrode strength. We compared the results obtained with a square and disk shape of the fibrotic patch with results from the one-dimensional simulations. Both, one- and two-dimensional simulations indicate that extracellular current application causes virtual electrodes at boundaries of fibrotic patches. A higher degree of fibrosis and larger patch size were associated with an increased strength of the virtual electrodes. Also, patch geometry affected the strength of the virtual electrodes. Our simulations suggest that increased fibroblast-myocyte coupling and intra-fibroblast conductivity reduce virtual electrode strength. However, experimental data to constrain these modeling parameters are limited and thus pinpointing the magnitude of the reduction will require further understanding of electrical coupling of fibroblasts in native cardiac tissues. We propose that the findings from our computational studies are important for development of patient-specific protocols for internal defibrillators.

## Introduction

Several types of cardiac arrhythmias are treated with external or implanted defibrillators, which are devices for application of electrical current to a patient's thorax or heart (Al-Khatib et al., 2017). The electrical currents create an electrical field in the heart, which is determined by the location and geometry of the electrodes, the magnitude and waveform of the applied current, and the distribution, composition and electrical properties of tissues. Targets of defibrillation are constituents of cardiac muscle tissues, i.e., the myocytes. These cells respond to an extracellular electrical field caused by a defibrillator with changes of their transmembrane voltage. Modulated by the electrophysiological state of the myocytes, their transmembrane voltage will exhibit negative or positive shifts due to the electrical field. Thus, defibrillation might cause hyperpolarization and depolarization of myocytes. Also, defibrillation might trigger or modulate action potentials.

Several theories for mechanisms of defibrillation of the heart have been developed (Dosdall et al., 2010). The critical mass theory suggests that defibrillation success requires myocyte depolarization in a sufficient mass of tissue. The upper limit of vulnerability theory proposes the existence of a minimal electrical stimulus strength above which ventricular fibrillation cannot be induced even for stimuli occurring during the vulnerable period of the cardiac cycle. The sawtooth hypothesis and the syncytial heterogeneity hypothesis explains defibrillation effects on the transmembrane voltage of myocytes distally from the electrodes based on microscopic discontinuities of the intracellular space (Fishler, 1998). Similarly, the virtual electrode theory explains defibrillation effects based on structural heterogeneities such as endo- and epicardial surfaces as well as blood vessels. Virtual electrodes are thought to underlie local shifts of transmembrane voltages and thus trigger action potentials and elicit wave fronts that interfere with fibrillation. Recent work using computational modeling focused on understanding virtual electrodes caused by vessels (Connolly et al., 2017a,b) and related curvature of the surface of tissue heterogeneities to myocyte depolarization and initiation of wave fronts (Bittihn et al., 2012).

It is well-established that patients with myocardial fibrosis profit from defibrillator implantation (Iles et al., 2011), but it has also been reported that patients exhibit an increased mortality if they receive defibrillation shocks (Cevik et al., 2009). Several cardiac diseases including fibrosis are associated with discontinuity of the intracellular space and microstructural heterogeneity. For instance, myocardial infarction leads to scar regions with a decreased volume ratio of myocytes, but increased volume ratios of the extracellular space and fibroblasts. Also, our recent studies on a rabbit model of myocardial infarction revealed patches of fibrotic tissue interspersed within working myocardium distal from the scar (Seidel et al., 2017). Furthermore, several types of fibrosis of cardiac tissue are characterized by local increases of extracellular space and fibroblasts. Currently, our knowledge on defibrillation effects in fibrotic tissues is sparse and we do not even know if understanding these effects can help with parameterization of defibrillation protocols. Based on virtual electrode theory, it is conceivable that fibrotic tissue will exhibit substantial effects in response to defibrillation. However, experimental and computational studies on this subject have not been performed.

Here, we investigated the effects of defibrillation on transmembrane voltages in fibrotic tissues. We applied an extended bidomain model of electrical conduction in cardiac tissue. In contrast to the conventional bidomain model, which describes myocyte and extracellular domains only, the extended model comprises a description of a fibroblast domain. We performed simulations in one-dimensional (1D) and two-dimensional (2D) models of cardiac tissues with fibrotic patches of variable sizes, tissue composition and myocyte-fibroblast electrical coupling. We measured shifts of the myocyte membrane voltage caused by application of strong extracellular currents to assess the strength of virtual electrodes.

## Methods

### Extended Bidomain Modeling of Cardiac Conduction

We utilized the extended bidomain model for computational simulations of defibrillation in 1D and 2D domains (Sachse et al., 2009). The model was developed based on the established bidomain model (Tung, 1978; Henriquez, 1993) and allows description of cardiac tissues comprising more than one cell species. The original extended bidomain model comprised two cell species, i.e., cardiac myocytes and fibroblasts. Intracellular domains for these two species together with the extracellular domain constitute three domains of interest.

The mathematical formulation of the extended bidomain model applies Kirchhoff's electrical current conservation law to myocardium (Plonsey and Barr, 2007). Here we followed the exposition in Sachse et al. (2009), which described the electrical dynamics of the myocardium in three “homogenized” continuous domains. In short, the expression of current conservation at any point in space mathematically translates into a Poisson equation for each of the three domains:

(1) $$\begin{array}{r} \nabla \cdot \left(\sigma_{myo}\nabla \phi_{myo}\right)=\;{-f}_{s,myo}+\;\beta_{myo,fib\;}I_{myo,fib}+\;\beta_{myo}{\;I}_{myo,e} \end{array}$$

(2) $$\begin{array}{r} \nabla \cdot \left(\sigma_{fib}\nabla \phi_{fib}\right)=\;{-f}_{s,fib}-\;\beta_{myo,fib\;}I_{myo,fib}+\;\beta_{fib\;}I_{fib,e} \end{array}$$

(3) $$\begin{array}{r} \nabla \cdot \left(\sigma_{e}\nabla \phi_{e}\right)=\;{-f}_{s,e}-\;\beta_{myo}{\;I}_{myo,e}-\;\beta_{fib}{\;I}_{fib,e} \end{array}$$

where σ_*myo*_, σ_*fib*_ and σ_*e*_ are the electrical conductivity tensors of the myocyte, fibroblast and extracellular domain, respectively, ϕ_*myo*_, ϕ_*fib*_ , and ϕ_*e*_ are the intracellular potential of the myocyte, fibroblast and extracellular domain, respectively, and *f*_*s,myo*_, *f*_*s,fib*_, and *f*_*s,e*_ (*A/m*^3^), are the stimulus current source densities for the myocyte, fibroblast and extracellular domain, respectively. Membrane voltage of myocytes *V*_*myo*_ and fibroblasts *V*_*fib*_ was defined as the difference of their intracellular and extracellular potential. The number of myocytes per unit volume β_*myo*_ and fibroblasts per unit volume β_*fib*_ (1/m^3^) were defined as:

(4) $$\begin{array}{r} \beta_{myo}=\frac{\;Vol_{myo}\;}{Vol_{myo,single}\;} \end{array}$$

(5) $$\begin{array}{r} \beta_{fib}=\frac{\;Vol_{fib}\;}{Vol_{fib,single}\;} \end{array}$$

with the volume fraction of myocytes *Vol*_*myo*_ and fibroblasts *Vol*_*fib*_ (dimensionless) as well as the individual volume for a myocyte *Vol*_*myo, single*_ and fibroblast *Vol*_*fib,single*_ (m^3^). Currents between the myocyte and extracellular, fibroblast and extracellular, and myocyte and fibroblast domains are identified by *I*_*myo,e*_, *I*_*fib,e*_, and *I*_*myo,fib*_*(A)*, respectively.

The current between the myocyte and fibroblast domain was defined as:

(6) $$\begin{array}{r} I_{myo,fib}=\frac{\phi_{myo}-\;\phi_{fib}\;}{R_{myo,fib}} \end{array}$$

where *R*_*myo,fib*_ is the resistance of gap junction channels (Ω). We defined the effective volume density β_*myo,fib*_ (1/m^3^) of electrical connections between fibroblasts and myocytes as:

(7) $$\begin{array}{r} \beta_{myo,fib}=\frac{\;Vol_{fib}\;}{Vol_{fib,single}\;} \end{array}$$

We assumed that the conductivity tensors (S/m) for each domain have a linear relationship with their respective volume fractions:

(8) $$\begin{array}{r} \sigma_{myo}=Vol_{myo\;}{\bar{\sigma }}_{myo} \end{array}$$

(9) $$\begin{array}{r} \sigma_{fib}=Vol_{fib\;}{\bar{\sigma }}_{fib} \end{array}$$

(10) $$\begin{array}{r} \sigma_{e}=Vol_{e\;}{\bar{\sigma }}_{e} \end{array}$$

where the tensors ${\bar{\sigma }}_{myo}$, ${\bar{\sigma }}_{fib}$ and ${\bar{\sigma }}_{e}$ described conductivity for volume ratios of 100%. In the subsequently described simulations, we vary ${\bar{\sigma }}_{fib}$ over a wide range of conductivities. We note that the range of σ_*fib*_ is smaller, due to scaling by the fibroblast volume fraction.

We applied a mathematical model of a rabbit ventricular myocyte (Mahajan et al., 2008) and a cardiac fibroblast (Sachse et al., 2008) to calculate the currents *I*_*myo,e*_ and *I*_*fib, e*_, respectively. Temperature of the fibroblast model was set to the same temperature as the myocyte model (308 K). We applied a temperature coefficient (Q10) of 2 to adjust rate coefficients of the time- and voltage dependent outward current of the fibroblast model.

### Implementation of Extended Bidomain Model

The extended bidomain model was implemented using the finite volume method and the programming language Fortran. The Portable, Extensible Toolkit for Scientific Computation (PETSc) (Balay et al., 2018) was used for parallelization and solving the Poisson equation. The myocyte (Cellml, 2008b) and fibroblast (Cellml, 2008a) models were implemented from CellML (Garny et al., 2008).

A simple forward Euler scheme with a fixed time step of 0.5 or 1 μs was applied to solve the time evolution of the model. The Poisson equation for the cellular domain was solved at each time step with a relative tolerance of 10^−12^. We applied the GMRES method, a member of the iterative Krylov methods that are implemented in PETSc. We used the additive Schwarz preconditioner in order to speed up the convergence for the iterative solver. The internal number of iterations for the Poisson solver for the 2D simulations was in the range of 50–100. This constituted the main burden of the numerical calculation. We checked for current conservation at each link of the discretized domain up to this precision (no numerical artifact of creation or annihilation of currents). In order to validate our numerical implementation, we also checked that the change in grid size discretization does not affect the results. We have compiled measured parameters in Tables S1–S3 to show that variation of spatial resolution in the range of 25–100 μm has only minor effects. We further evaluated this new implementation of the extended bidomain model using our prior implementation (Sachse et al., 2009; Seemann et al., 2010). Modifications of the prior implementation were performed to account for discontinuity in the material properties of the cardiac tissue at the interface between the control and fibrotic regions.

### Setup of 1D Simulations

**Figure 1:**
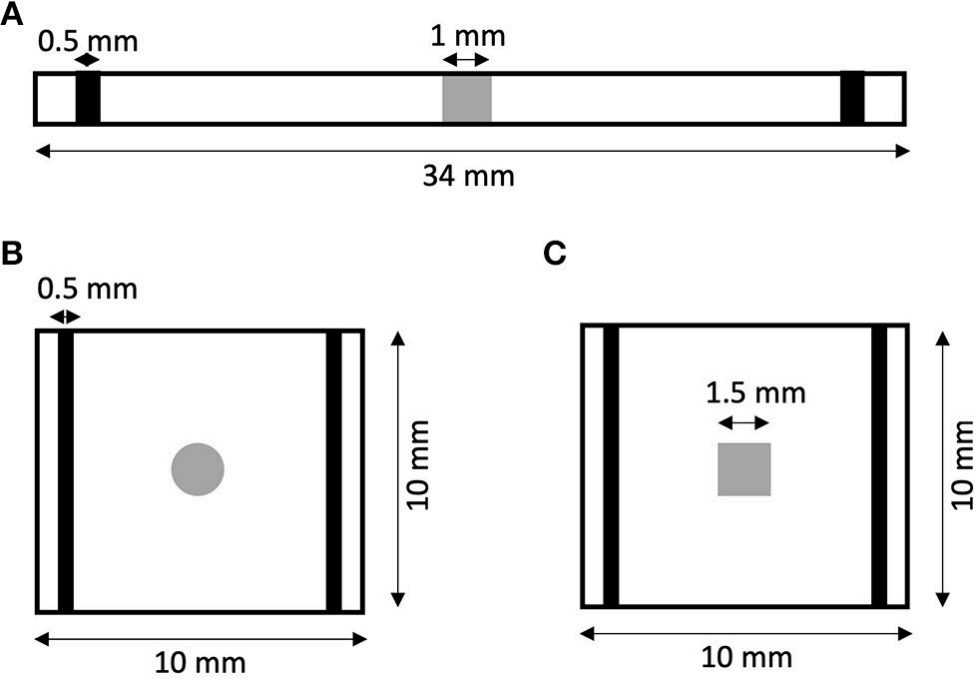
Geometry of **(A)** 1D and **(B,C)** 2D models of rabbit ventricular tissue with central fibrotic patch. The sites of extracellular current application are marked in black. Fibrotic patches are shown in grey.

We created 1D models of normal rabbit ventricular myocardium with an embedded central fibrotic patch (Figure 1A). The model was discretized with a spatial resolution of 50 or 100 μm. The patch exhibited differences in volume fractions of extracellular space, myocytes and fibroblasts measured in our previous work of fibrotic tissue (Greiner et al., 2018). Two different types of patches representing average and large fibrosis were configured (Table 1). We varied *R*_*myo,fib*_ and ${\bar{\sigma }}_{fib}$ in simulations. The simulations started with applying 9 depolarizing myocyte membrane currents at the location 32.5–33 mm of the domain with an amplitude of 20 μA/cm^2^ and a duration of 5 ms at a frequency of 4 Hz. At 250 ms after the last stimulus, extracellular currents were applied at 1–1.5 mm and 32.5–33 mm of the domain with an amplitude of 0.7 × 10^5^ and −0.7 × 10^5^ A/m^3^, respectively. These injected currents created a nearly uniform electric field between the two sites of magnitude *E* = 0.546 V/cm (Figure S1). This electric field, in turn, was responsible for the generation of the virtual electrodes at the left (referred as point 1) and right (point 2) boundary of the fibrotic patch.

**Table 1:** Parameters of extended bidomain model.

**Parameter**	**Symbol**	**Region**	**Value**
Extracellular volume	*Vol*_*e*_	Control	32
fraction (%)		Average fibrosis	43
		Large fibrosis	60
Myocyte volume	*Vol*_*myo*_	Control	65
fraction (%)		Average fibrosis	47
		Large fibrosis	20
Fibroblast volume	*Vol*_*fib*_	Control	3
fraction (%)		Average fibrosis	10
		Large fibrosis	20
Extracellular conductivity (S/m)	${\bar{\sigma }}_{e}$		1
Intra-myocyte conductivity (S/m)	${\bar{\sigma }}_{myo}$		0.5
Intra-fibroblast conductivity (S/m)	${\bar{\sigma }}_{fib}$		0, 0.1, 0.2, 0.5
Myocyte-fibroblast coupling resistance (MΩ)	*R*_*myo,fib*_		1–10^6^
Volume of single myocyte (pL)	*Vol*_*myo,single*_		10.299
Volume of single fibroblast (pL)	*Vol*_*fib,single*_		0.268
Membrane capacitance per unit area (μF/cm^2^)			1
Myocyte surface to volume ratio (cm^−1^)			2,500
Fibroblast surface to volume ratio (cm^−1^)			16,800

In order to measure virtual electrode strengths, we monitored the difference in the membrane voltage of the myocytes *V*_*myo*_ and fibroblasts *V*_*fib*_ at patch boundaries during the application of extracellular current with respect to the membrane voltages before current application. *V*_*myo*_ was not a monotonic function of time during the current application (Figure S2). Therefore, we reported the largest absolute value (maintaining its sign) of the monitored difference during the current application as a measure of the local virtual electrode strength. In addition, we quantified space constants λ_myo_ and λ_fib_ by fitting the spatial profile of *V*_*myo*_ and *V*_*fib*_, respectively, at and proximal to the boundaries of the patch.

### Setup of 2D Simulations

We generated 2D models of normal rabbit ventricular myocardium with an embedded fibrotic patch of square and disk-like shapes (Figures 1B,C). The square side length and the disk diameter were both set to 1.5 mm. We discretized the model with a spatial resolution of 50 μm in x and y direction. The computational domain was 1 cm x 1 cm in size. We applied four intracellular stimuli at 9–9.5 mm along the length and entire width with an amplitude of 20 μA/cm^2^ for 5 ms at a frequency of 4 Hz. Two hundred and fifty milliseconds after the last stimulation, extracellular currents were applied at 0.5–1 mm and 9–9.5 mm along the length and throughout the width with an amplitude of 0.7 × 10^5^ and −0.7 × 10^5^ A/m^3^, respectively. These injected currents created a nearly uniform electric field between the two electrodes of magnitude 0.530 V/cm.

### Data Analysis

Effects of patch length *W*_*p*_ on Δ*V*_*myo*_ were analyzed using the fitting function:

(11) $$\begin{array}{r} \Delta V_{myo}=a\left(1-e^{-\frac{W_{p}}{b}}\right) \end{array}$$

with the parameters a and b. We used a different fitting function for Δ*V*_*fib*_, because it was not always crossing the origin of axes:

(12) $$\begin{array}{r} \Delta V_{fib}=c-ae^{-\frac{W_{p}}{b}} \end{array}$$

with the parameters a, b and c. We measured the fit quality using the adjusted *R*^2^.

## Results

### 1D Simulations

**Figure 2:**
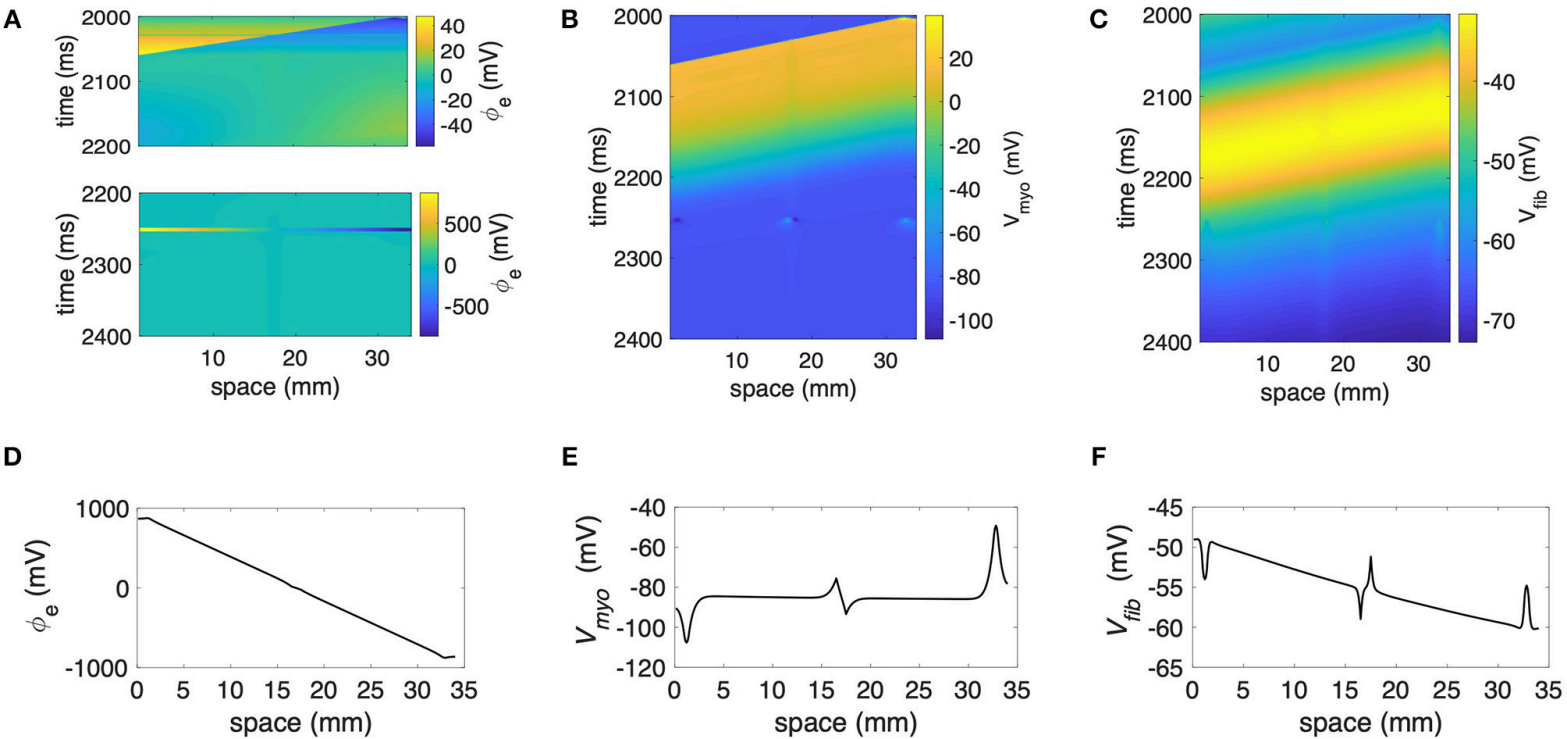
Representative plots of **(A)** ϕ_*e*_, **(B)** *V*_*myo*_, and **(C)** *V*_*fib*_for intracellular pacing and subsequent extracellular current application in the 1D model with the average fibrosis patch, ${\bar{\sigma }}_{fib}$ of 0.1 S/m and *R*_*myo,fib*_ of 40 GΩ. Spatial distribution of **(D)** ϕ_*e*_, **(E)** *V*_*myo*_, and **(F)** *V*_*fib*_ at the end of application of extracellular currents. The extracellular current led to alterations of *V*_*myo*_ and *V*_*fib*_ not only at the application site, but also at the left and right border of the fibrotic patch.

We performed numerical simulations with the 1D extended bidomain model of control tissue with a central patch configured with parameters for average fibrosis (Figure 1A). The model was discretized with a spatial resolution of 100 μm. In Figures 2A–C, we present the results of a simulation of a propagating wave initiated by intracellular stimulation followed by application of extracellular current. The model parameters were *R*_*myo,fib*_ = 4 × 10^4^ MΩ and ${\bar{\sigma }}_{fib}=0.1$ S/m. After scaling with the fibroblast volume fraction (Equation 9) in control tissue (3%) and patch (10%), σ_*fib*_ amounted to 0.003 and 0.01 S/m, respectively. In this case, the propagation of action potentials was only marginally affected by the fibrotic patch. Extracellular current altered ϕ_*e*_ as well as *V*_*myo*_ and *V*_*fib*_ at the application sites and the borders of the fibrotic patch.

We present the spatial distribution of ϕ_*e*_, *V*_*myo*_, and *V*_*fib*_ at the end (*t* = 2.255 s) of current application in Figures 2D–F, respectively. Due to current application, *V*_*myo*_ at the left border of the patch was increased from −84.95 mV before current application to −75.59 mV at the end of the current application. *V*_*fib*_ was reduced from −54.05 mV before current application to −58.97 mV at the end of the current application. At the right border of the patch *V*_*myo*_ was decreased from −85.00 to −93.44 mV. *V*_*fib*_ was increased from −54.21 to −51.18 mV. We determined the electric field for average and large fibrosis during the current application (Figures S1A,B, respectively). The spatial variation outside of the region for current application was small.

**Figure 3:**
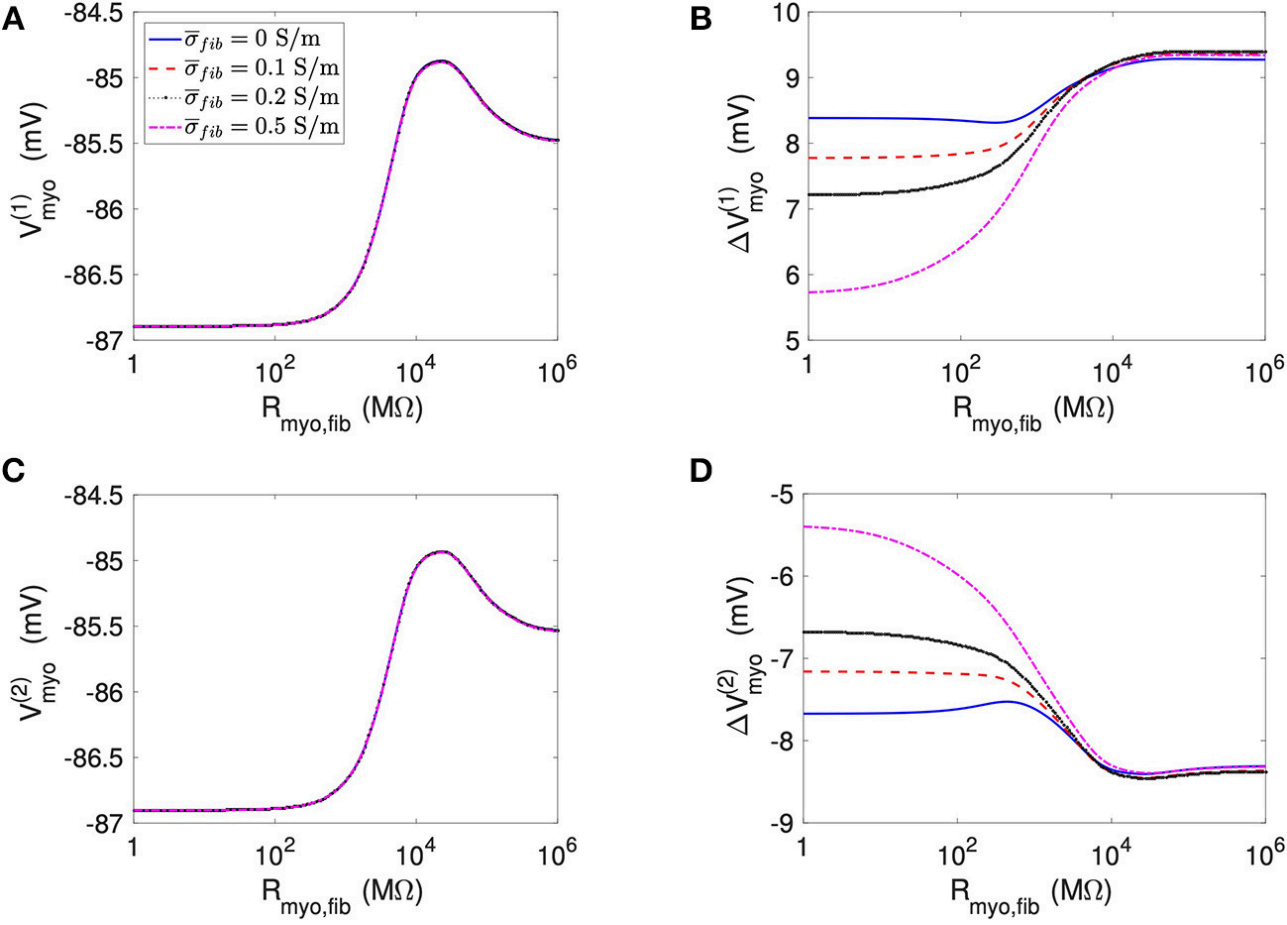
Measurement of effects of extracellular current application on *V*_*myo*_ using the 1D model with the average fibrosis patch. Before application of extracellular currents, *V*_*myo*_ at the **(A)** left and **(C)** right border of the fibrotic patch was marginally affected by *R*_*myo,fib*_ and ${\bar{\sigma }}_{fib}$. **(B)** At the end of current application, the left patch border exhibited a positive Δ*V*_*myo*_. **(D)** In contrast, the right patch border exhibited a negative Δ*V*_*myo*_.

We applied the 1D model in a series of simulations varying *R*_*myo,fib*_ and ${\bar{\sigma }}_{fib}$. Example simulated ϕ_*e*_, *V*_*myo*_, and *V*_*fib*_ are presented in Figure S3. Before current application, the biphasic relationship of *V*_*myo*_ with *R*_*myo,fib*_ was nearly identical on both boundaries of the patch (Figures 3A,C). Also, variation of ${\bar{\sigma }}_{fib}$ did not affect *V*_*myo*_. After current application, the left border of the patch exhibited increased *V*_*myo*_, measured as $\Delta V_{myo}^{\left(1\right)}$, dependent on *R*_*myo,fib*_ and ${\bar{\sigma }}_{fib}$ (Figure 3B). At the right border of the patch, the reduction of *V*_*myo*_, measured as $\Delta V_{myo}^{\left(2\right)}$, was dependent on *R*_*myo,fib*_ and ${\bar{\sigma }}_{fib}$ (Figure 3D). At both borders of the patch, effects on *V*_*myo*_ were of similar magnitude for all ${\bar{\sigma }}_{fib}$ for large *R*_*myo,fib*_.

**Figure 4:**
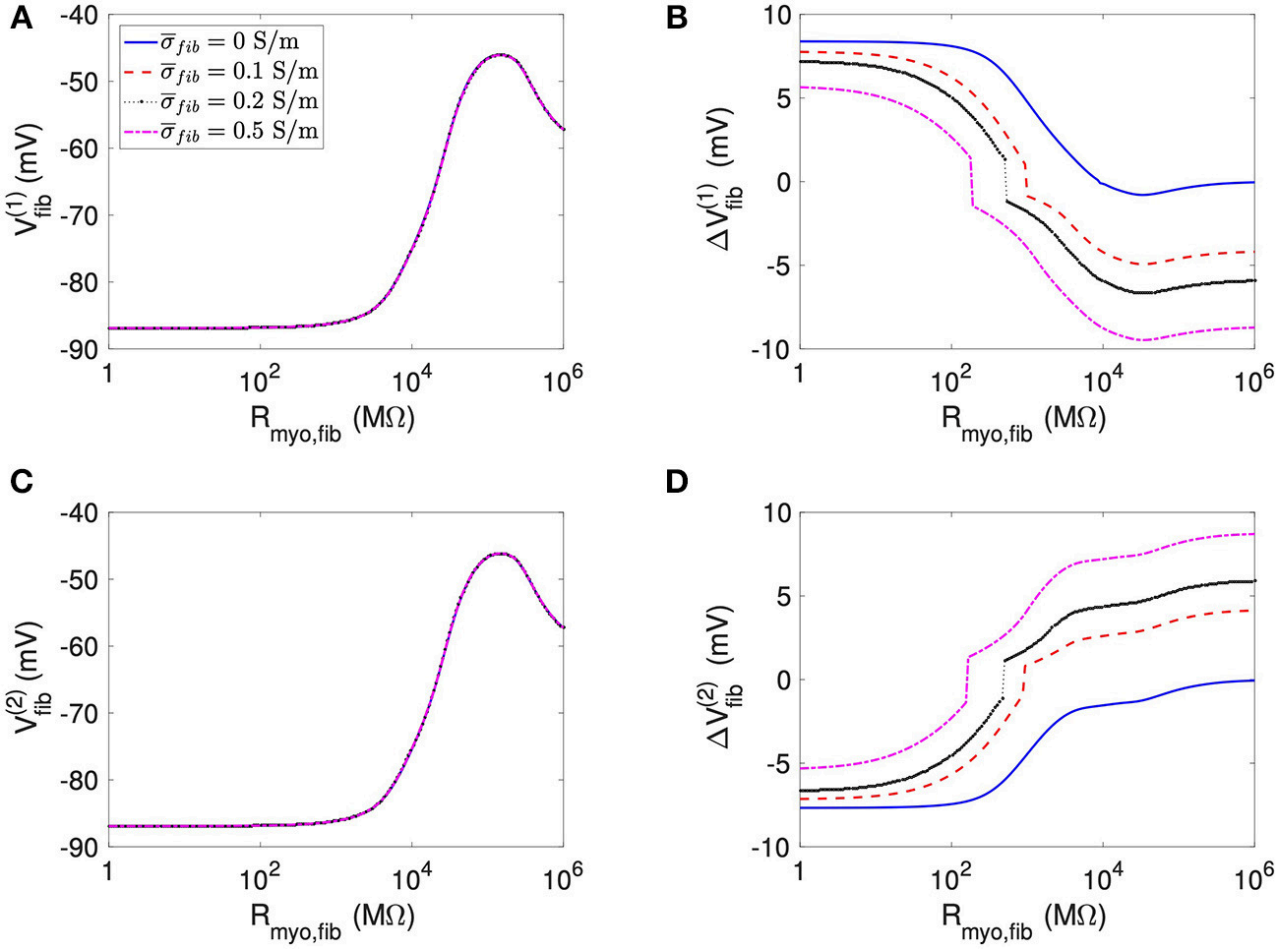
Measurement of effects of extracellular current application on *V*_*fib*_ using the 1D model with the average fibrosis patch. Before application of extracellular currents, *V*_*fib*_ at the **(A)** left and **(C)** right border of the fibrotic patch was strongly affected by *R*_*myo,fib*_ and ${\bar{\sigma }}_{fib}$. At the end of current application, the **(B)** left and **(D)** right border exhibited a Δ*V*_*fib*_ with magnitude and sign modulated by *R*_*myo,fib*_ and ${\bar{\sigma }}_{fib}$.

We present corresponding measures on *V*_*fib*_ in Figure 4. The biphasic relationship of *V*_*fib*_ with *R*_*myo,fib*_ before application of extracellular current (Figures 4A,C) was similar to the relationship of *V*_*myo*_ with *R*_*myo,fib*_. For low *R*_*myo,fib*_ (high myocyte-fibroblast coupling), *V*_*fib*_ was similar to the resting *V*_*myo*_ (Figures 3A,C). For high *R*_*myo,fib*_ (low myocyte-fibroblast coupling), *V*_*fib*_was close to the membrane voltage of isolated fibroblasts, i.e., ~58 mV (Shibukawa et al., 2005; Sachse et al., 2008). Similar as myocytes, fibroblasts at the borders of the patch exhibit alterations of their membrane voltage, $\Delta V_{fib}^{\left(1\right)}$ and $\Delta V_{fib}^{\left(2\right)}$, dependent on *R*_*myo,fib*_ and ${\bar{\sigma }}_{fib}$ in response to current application (Figures 4B,D). For low *R*_*myo,fib*_, Δ*V*_*fib*_ was roughly similar to the Δ*V*_*myo*_ at both borders. In contrast, for high *R*_*myo,fib*_, Δ*V*_*fib*_ exhibited a reverse sign vs. the corresponding Δ*V*_*myo*_ at both borders. This results in the discontinuity in Δ*V*_*fib*_ for *R*_*myo,fib*_ of 100–1,000 MΩ (Figures 4B,D).

**Figure 5:**
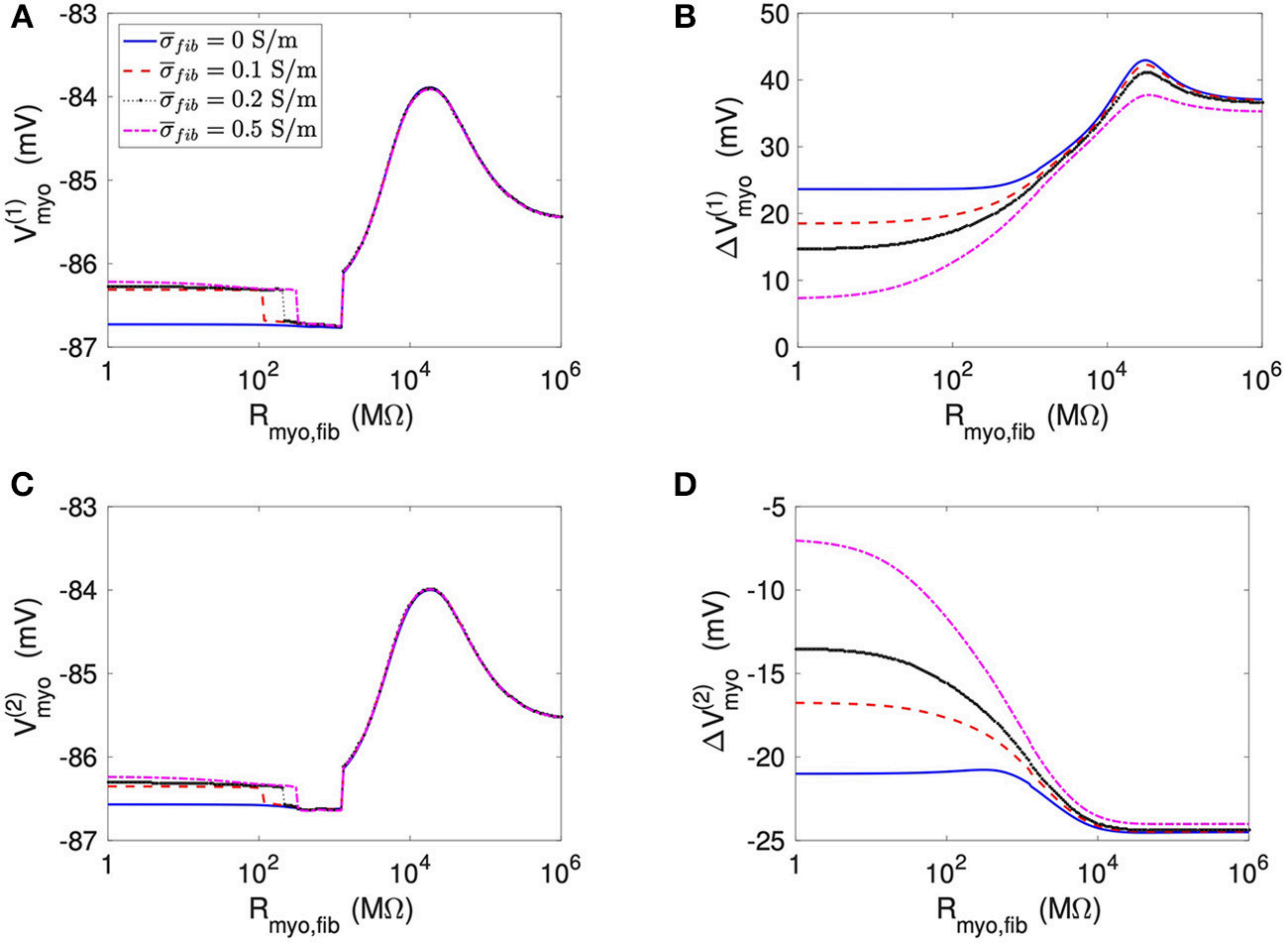
Measurement of effects of extracellular current application on *V*_*myo*_ using the 1D model with the large fibrosis patch. Before application of extracellular currents, *V*_*myo*_ at the **(A)** left and **(C)** right border of the fibrotic patch was marginally affected by *R*_*myo,fib*_ and ${\bar{\sigma }}_{fib}$. **(B)** At the end of current application, the left patch border exhibited a positive Δ*V*_*myo*_. **(D)** In contrast, the right patch border exhibited a negative Δ*V*_*myo*_.

**Figure 6:**
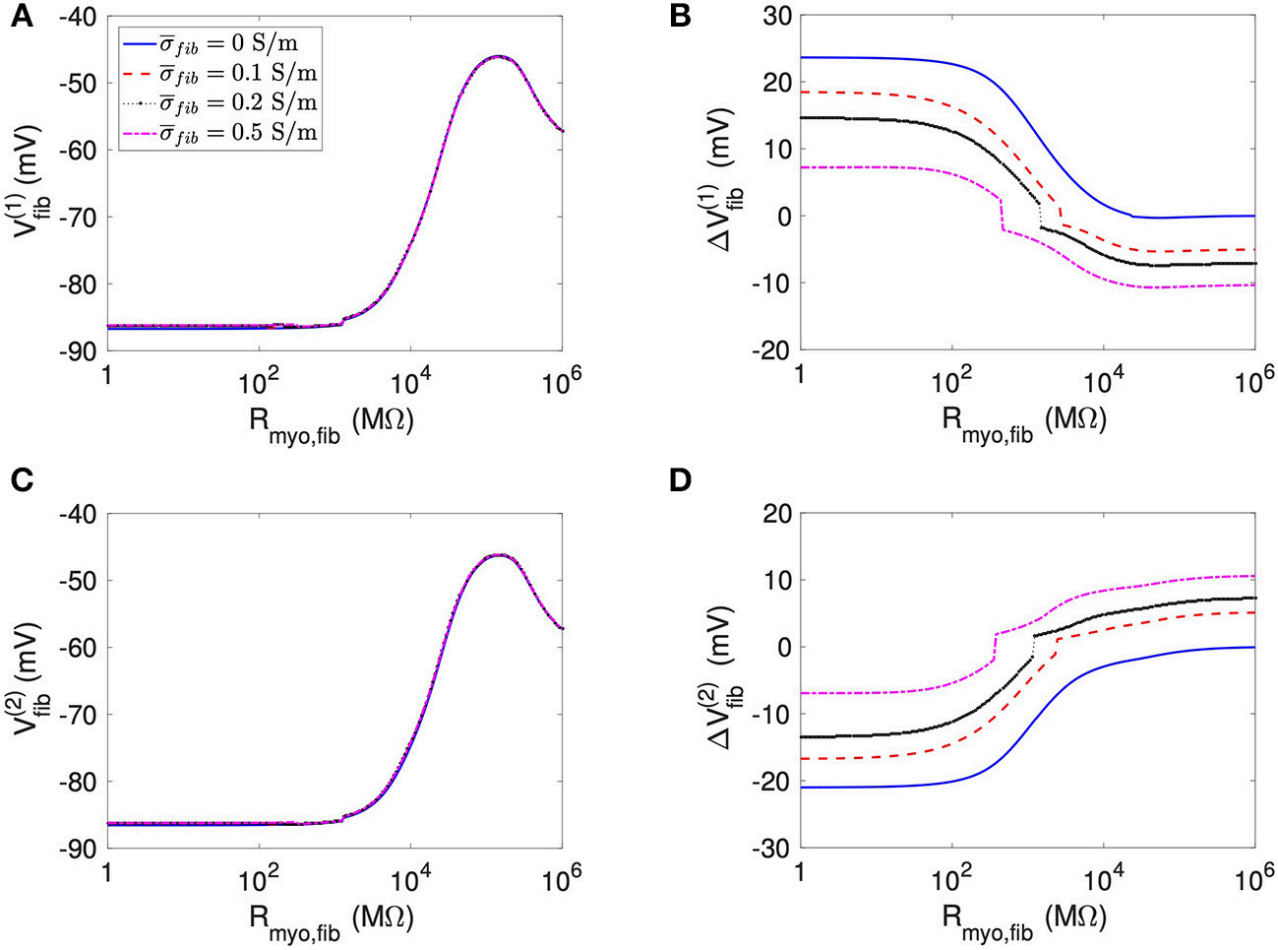
Measurement of effects of extracellular current application on *V*_*fib*_ using the 1D model with the large fibrosis patch. Before application of extracellular currents, *V*_*fib*_ at the **(A)** left and **(C)** right border of the fibrotic patch was strongly affected by *R*_*myo,fib*_ and ${\bar{\sigma }}_{fib}$. At the end of current application, the **(B)** left and **(D)** right border exhibited a Δ*V*_*fib*_ with magnitude and sign modulated by *R*_*myo,fib*_ and ${\bar{\sigma }}_{fib}$.

**Figure 7:**
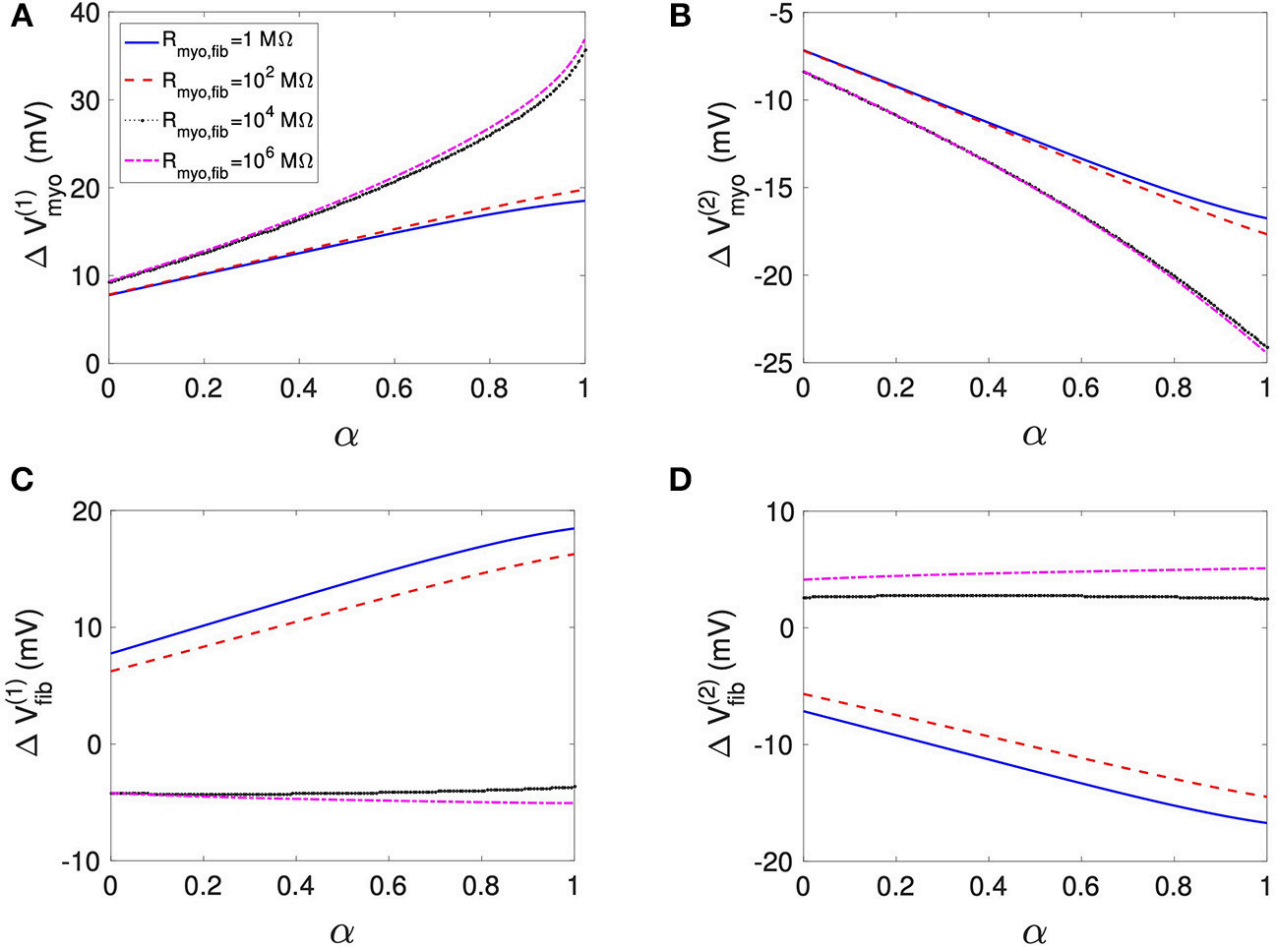
Assessment of effects of extracellular current application on *V*_*myo*_ and *V*_*fib*_ simulated in 1D models with a patch of varying degrees of fibrosis. The degree of fibrosis ranged from average (α = 0) to large (α = 1). We also varied *R*_*myo,fib*_. ${\bar{\sigma }}_{fib}$ was set to 0.1 S/m. The effects on the magnitude of Δ*V*_*myo*_ at the **(A)** left and **(B)** right boundary increased with the degree of fibrosis. Similarly, effects of Δ*V*_*fib*_ at the **(C)** left and **(D)** right boundary increased with the degree of fibrosis for small *R*_*myo,fib*_. Marginal effects were present for larger *R*_*myo,fib*_.

Using the same geometrical model and experimental protocol, we also performed simulations using a patch with large fibrosis (Figures 5, 6). While the results were qualitatively similar as for the average case of fibrosis described above, we note important differences. In particular, the higher degree of fibrosis was associated with a higher magnitude of Δ*V*_*myo*_ and Δ*V*_*fib*_. We observed conduction block for *R*_*myo,fib*_ of 100–1,000 MΩ (Figures 5A,C). We confirmed that the magnitude Δ*V*_*myo*_ is a monotonously increasing function of the degree of fibrosis (Figures 7A,B). The parameter α described the variation between the low degree of fibrosis (α = 0) to the high degree of fibrosis (α = 1). In the range of settings that we have studied, the variation of Δ*V*_*myo*_ with α was roughly linear. Similarly, for low *R*_*myo,fib*_ the magnitude Δ*V*_*fib*_ was a monotonously increasing function of α (Figures 7C,D). For high *R*_*myo,fib*_, the magnitude Δ*V*_*fib*_ was approximately constant for α between 0 and 1.

**Figure 8:**
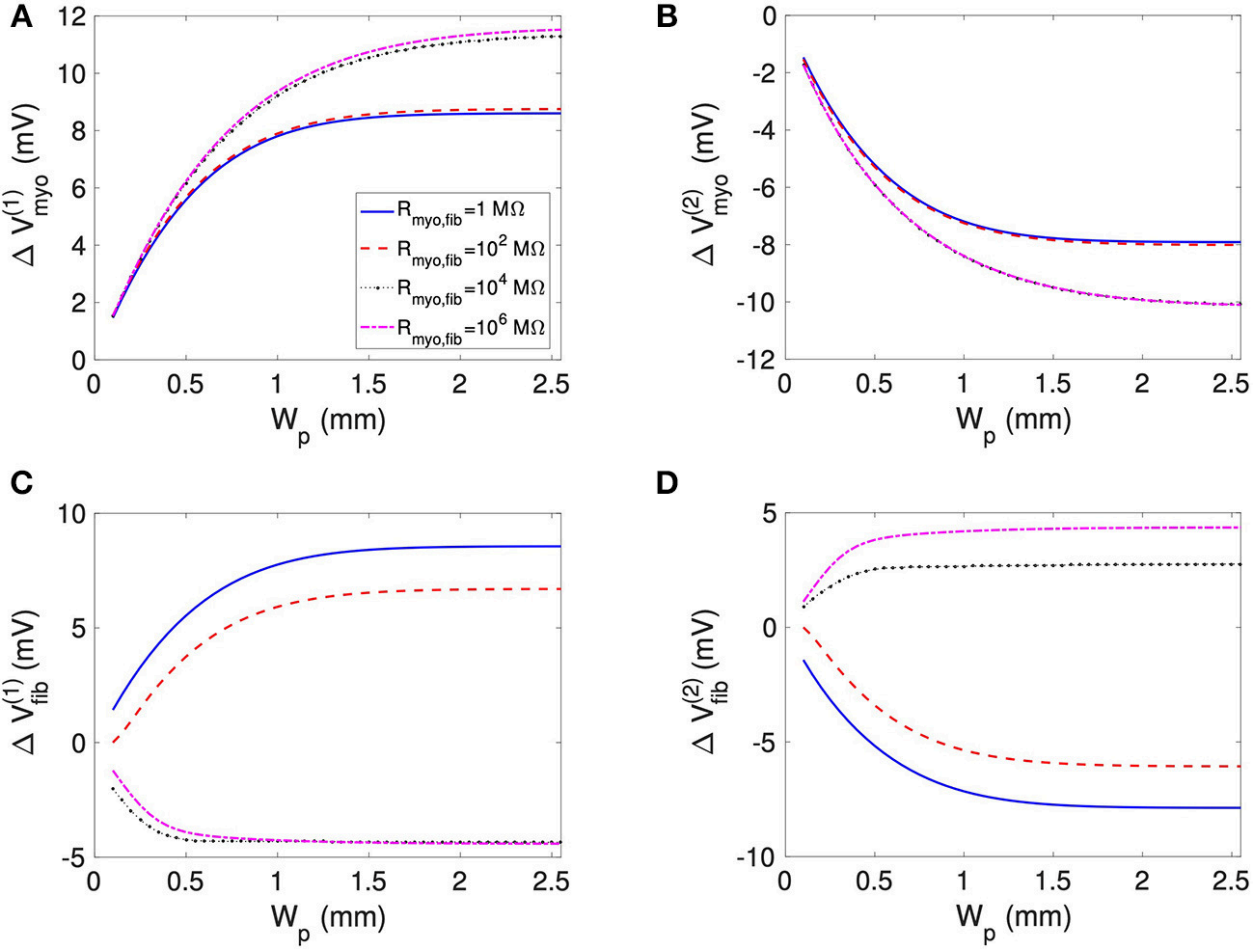
Effects of patch length W_p_ on **(A,B)** *V*_*myo*_ and **(C,D)** *V*_*fib*_ at the boundary of the patch in response to application of the extracellular current. We varied *R*_*myo,fib*_, but ${\bar{\sigma }}_{fib}$ was set on 0.1 S/m. For small W_p_, *V*_*myo*_, and *V*_*fib*_ were greatly reduced.

We assessed the influence of patch length on virtual electrode strengths (Figure 8). The simulations were performed with a spatial resolution of 50 μm in order to explore smaller patch lengths. We present fit parameters of Δ*V*_*myo*_ and Δ*V*_*fib*_ in Table 2. The patch length was a major modulator of virtual electrode strengths. The larger the patch, the higher the effect on membrane voltages. However, there is a limit to this phenomenon, with the effect tapering off for longer patches. We assessed the length scale, which depends on the coupling between the myocyte and fibroblast domain through the coupling parameter *R*_*myo,fib*_. For large *R*_*myo,fib*_, the spatial length scales for the myocytes and fibroblasts correspond to their respective space constants λ_myo_ and λ_fib_. In contrast, for low *R*_*myo,fib*_ the spatial length scales were the same for the two species due to the coupling and settled at an intermediate value between λ_myo_ and λ_fib_. Indeed, larger difference of the Δ*V*_*myo*_ and Δ*V*_*fib*_ were at the sub-millimeter scale (Figure 8), which is in agreement with the estimated values for of λ_myo_ and λ_fib_ (Tables S1–S3).

**Table 2:** Relationships of membrane voltage changes and W_p_ according to the fitting functions.

	**R_**myo,fib**_ (MΩ)**	**1**	**10^**2**^**	**10^**4**^**	**10^**6**^**
${\Delta \text{V}}_{\text{m}y\text{o}}^{\left(1\right)}$	a (mV)	8.778 (8.723, 8.833)	8.907 (8.862, 8.953)	11.65 (11.59, 11.71)	11.9 (11.84, 11.96)
	b (mm)	0.485 (0.473, 0.497)	0.4843 (0.4747, 0.494)	0.654 (0.6438,0.664)	0.6616 (0.651, 0.672)
	Adj. *R*^2^	0.9965	0.9977	0.999	0.9989
${\Delta \text{V}}_{\text{m}y\text{o}}^{\left(2\right)}$	a (mV)	−8.049 (−8.087, −8.012)	−8.126 (−8.154, −8.097)	−10.24 (−10.25, −10.22)	−10.25 (−10.26, −10.23)
	b (mm)	0.4702 (0.4614, 0.479)	0.4671 (0.4606, 0.4737)	0.5756 (0.5725, 0.5787)	0.581 (0.578, 0.584)
	Adj. *R*^2^	0.9979	0.9988	0.999	0.9999
$\Delta {\text{V}}_{\text{f}i\text{b}}^{\left(1\right)}$	a (mV)	9.311 (9.171, 9.451)	8.965 (8.784, 9.146)	−4.682 (−4.902, −4.462)	−5.064 (−5.225, −4.903)
	b (mm)	0.447 (0.436, 0.4587)	0.455 (0.4396, 0.4709)	0.1521 (0.1454, 0.1588)	0.2181 (0.2103, 0.2259)
	c (mV)	8.677 (8.64, 8.714)	6.82 (6.771, 6.869)	−4.342 (−4.353, −4.33)	−4.374 (−4.389, −4.359)
	Adj. *R*^2^	0.9985	0.9973	0.9944	0.9964
$\Delta {\text{V}}_{\text{f}i\text{b}}^{\left(2\right)}$	a (mV)	−8.379 (−8.49, −8.26)	−8.106 (−8.265, −7.946)	3.136 (3.003, 3.269)	5.05 (4.887, 5.213)
	b (mm)	0.445 (0.435, 0.456)	0.454 (0.439, 0.4693)	0.1985 (0.1895, 0.2075)	0.2242 (0.216, 0.2324)
	c (mV)	−7.97 (−8.004, −7.94)	−6.17 (−6.213, −6.127)	2.728 (2.717, 2.738)	4.315 (4.299, 4.33)
	Adj. *R*^2^	0.9988	0.9975	0.9941	0.9962

*The values given in parentheses indicate a 95 % confidence interval for the parameter estimates*.

### 2D Simulations

We investigated virtual electrodes in a 2D spatial domain of control tissue with central patches configured with parameters for average fibrosis (Figures 1B,C). Because we know from the 1D simulations that effects of the patch on Δ*V*_*myo*_ and Δ*V*_*fib*_ are larger at the millimeter than sub-millimeter scale, we set the patches (square and disk) to a size of 1.5 mm.

**Figure 9:**
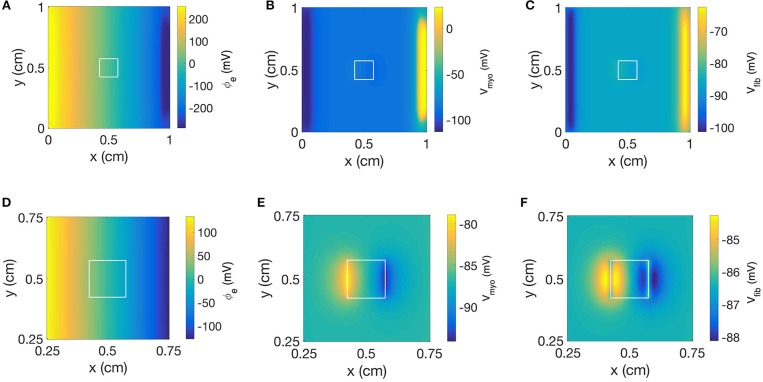
Spatial distribution of **(A)** ϕ_*e*_, **(B)** *V*_*myo*_, and **(C)** *V*_*fib*_ at the end of extracellular current application in the 2D model with the square-shaped average fibrosis patch, ${\bar{\sigma }}_{fib}$ of 0.1 S/m and *R*_*myo,fib*_ of 1 GΩ. Close-ups of the spatial distribution for **(D)** ϕ_*e*_, **(E)** *V*_*myo*_, and **(F)** *V*_*fib*_. The extracellular current led to alterations of *V*_*myo*_ and *V*_*fib*_ not only at the application site, but also at the left and right border of the patch.

Figure 9 displays the 3 main fields at the end of the extracellular current application in a model with a square-shaped patch, ${\bar{\sigma }}_{fib}$ of 0.1 S/m and *R*_*myo,fib*_ of 1 GΩ. The injected currents at the electrode generated a fairly linear distribution for ϕ_*e*_ (Figures 9A,D), which implies a quasi-uniform electric field. Only close to the cathode (x≈9 mm) the field was distorted by a nascent wave initiated at the cathode location. Closer inspection revealed that the field is really uniform close to the patch (Figure 9D). The corresponding spatial distribution of *V*_*myo*_ for the same time shows the virtual electrodes at the two edges of the square that are perpendicular to the applied field (Figures 9B,E). The edge corresponding to the small value of x exhibited a depolarization pattern (equivalent to point 1 in the 1D setting). On the contrary the other edge exhibited a hyperpolarization pattern (equivalent to point 2 in the 1D setting). Figures 9C,F display the field for *V*_*fib*_. In this example, *R*_*myo, fib*_ was set to 1 GΩ, which is the value for which the coupling between the myocytes and the fibroblast cancels out the effect of the conductivity heterogeneities at the patch boundaries. According to the theory (see section Discussion), we expect to observe hyperpolarization for *V*_*fib*_ at point 1 and depolarization at point 2, which is the opposite of what happens to *V*_*myo*_. However, the coupling term between the two domains caused that the *V*_*myo*_ is pulling the *V*_*fib*_ in the same direction. At the very edge of the patch (Figure 9F) the two effects cancel out.

**Figure 10:**
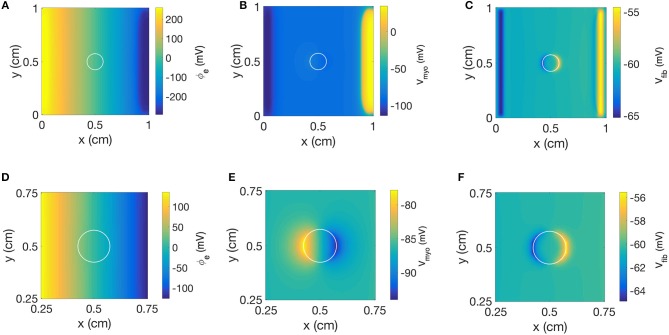
Spatial distribution of **(A)** ϕ_*e*_, **(B)** *V*_*myo*_, and **(C)** *V*_*fib*_ at the end of extracellular current application in the 2D model with the disk-shaped average fibrosis patch, ${\bar{\sigma }}_{fib}$ of 0.1 S/m and *R*_*myo,fib*_ of 1 TΩ. Close-ups of the spatial distribution for **(D)** ϕ_*e*_, **(E)** *V*_*myo*_, and **(F)** *V*_*fib*_ at the end of application of extracellular currents. The extracellular current led to alterations of *V*_*myo*_ and *V*_*fib*_ not only at the application site, but also at the left and right border of the patch.

We repeated the simulation using a disk-shaped patch with *R*_*myo,fib*_ = 1 TΩ, which represents negligible coupling between *V*_*fib*_ and *V*_*myo*_ (Figure 10). The simulation yielded a linear distribution for ϕ_*e*_ and the simulated electric field was almost identical as the field obtained with the square patch (Figures 10A,D). In Figures 10B,E we present the corresponding spatial distribution of *V*_*myo*_. As before, we observed the two virtual electrodes at the edges of the patch. Due to the disk-shaped patch, the virtual electrodes were not so sharp and appeared diffuse also in the parallel direction of the electric field. Figures 10C,F confirmed that polarities of Δ*V*_*fib*_ are indeed opposite to the Δ*V*_*myo*_ at the patch boundary. Also, the spatial scale for the virtual electrode was much smaller than the virtual electrodes for the *V*_*myo*_ field, which agrees with the corresponding values for the space constants associated with the two fields.

**Figure 11:**
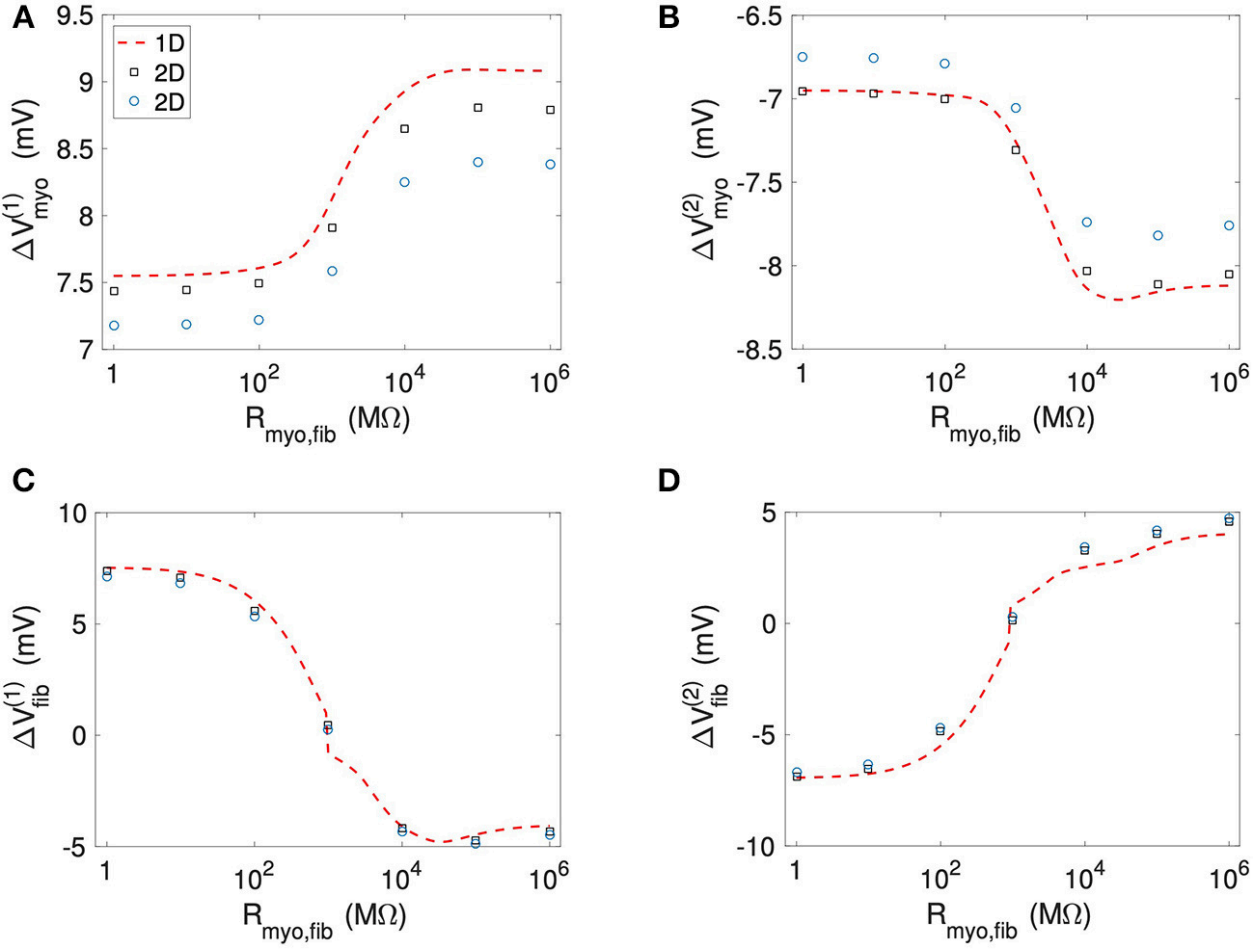
Comparison of **(A,B)** *V*_*myo*_ and **(C,D)** *V*_*fib*_ from the 1D simulations and the 2D simulations with disk and square patch geometries. The square and circle symbols refer to the results for the respective square and disk patch geometry.

Figure 11 and Table S4 present quantitative measures for the virtual electrode strengths for the two patch geometries. We report the results for Δ*V*_*myo*_ and Δ*V*_*fib*_ at both locations of the patch boundaries as a function of *R*_*myo, fib*_. We compared them with the corresponding values obtained for the 1D case. Using bidomain models it has been demonstrated that convex shape of boundaries leads to lower activation of the membrane (Entcheva et al., 1998, 1999; Pumir and Krinsky, 1999; Bittihn et al., 2012). This was confirmed in our simulation for Δ*V*_*myo*_ where the virtual electrode strengths were lower for the disk than for the square. However, the difference between the two geometries is rather minute. We measured an approximate 4% difference of Δ*V*_*myo*_ for low values of the *R*_*myo,fib*_ and an approximate 5% difference for high values of the *R*_*myo,fib*_. Also, Δ*V*_*myo*_ for the square-shaped were higher than for the disk-shaped patch, and close to the 1D patch geometry. Δ*V*_*fib*_ for the 1D and the two 2D geometries were similar (Figures 11C,D). For Δ*V*_*fib*_, the influence of patch geometry was less pronounced.

## Discussion

Our computational study provided insights into the relationship between parameters of fibrotic patches and the strength of virtual electrodes caused by defibrillation. We applied the extended bidomain model to reflect that cardiac tissues comprise fibroblasts beyond myocytes. Our study showed that an increased degree of fibrosis and an increased size of the fibrotic patch cause an increased strength of virtual electrodes. Also, we found that the composition of tissue heterogeneities is a modulator of virtual electrode strength. Increased electrical coupling of myocytes with fibroblasts reduced the virtual electrode strength. Intra-fibroblast coupling reduced virtual electrodes in case of high myocyte-fibroblast coupling.

We explain our findings of depolarization and hyperpolarization of membranes during the application of the extracellular currents at the boundaries of the fibrotic patch based on reformulation of the equations for the extended bidomain. The equations can be rewritten in the following simple form for numerical solution see Equations (17–20) in Sachse et al. (2009):

(13) $$\begin{array}{l} \frac{\partial V_{myo}}{\partial t}=\frac{1}{C_{myo}}(\nabla \cdot \left(\sigma_{myo}\nabla V_{myo}\right)+\nabla \cdot \left(\sigma_{myo}\nabla \phi_{e}\right)\\ +\cdots ) \end{array}$$

(14) $$\begin{array}{l} \frac{\partial V_{fib}}{\partial t}=\frac{1}{C_{fib}}(\nabla \cdot \left(\sigma_{fib}\nabla V_{fib}\right)+\nabla \cdot \left(\sigma_{fib}\nabla \phi_{e}\right)\\ +\cdots ) \end{array}$$

(15) $$\begin{array}{r} \nabla \cdot \left\{\left({\sigma_{e}+\sigma_{myo}+\sigma }_{fib}\nabla \phi_{e}\right)\right\}=\;{-f}_{e}-\nabla \cdot \left(\sigma_{myo}\nabla V_{myo}\right)\\ -\nabla \cdot \left(\sigma_{fib}\nabla V_{fib}\right) \end{array}$$

with the injected current *f*_*e*_ due to extracellular current application, the myocyte membrane capacitance *C*_*myo*_ and the fibroblast membrane capacitance *C*_*fib*_. Equations (13) and (14) are parabolic equations for the time evolution of the myocytes and fibroblast membrane voltage. Equation (15) is the Poisson equation for calculating the extracellular potential. In these equations, ⋯  indicates terms that are not of interest for our initial discussion and hence omitted.

During the current application, we injected positive charge at the electrode located at 1–1.5 mm and negative charge of the same magnitude at the electrode located at 32.5–33 mm of the extracellular domain. This current injection produced a rather uniform gradient of ϕ_*e*_ (Figure 2). This corresponds to a quasi-uniform E in the system (Figure S1):

(16) $$\begin{array}{r} E=\;-\;\nabla \phi_{e} \end{array}$$

With our computational set-up, *E* is oriented in the same direction as the x-axis. *E* together with the discontinuity of the conductivity at the patch boundary is responsible for the initial changes of the membrane voltage during the current application. This can be shown by decomposing the second right hand term of Equation (13) into:

(17) $$\begin{array}{r} \frac{\partial V_{myo}}{\partial t}\propto \;-\left(\nabla \sigma_{myo}\right)\cdot E+\sigma_{myo}\nabla^{2}\phi_{e} \end{array}$$

This represents the generalized activating function previously described to predict the distribution of virtual electrodes (Sobie et al., 1997). The crucial term in determining the direction of the membrane voltage change during the current application is the first right hand side term. If ∇σ_*myo*_ is oriented in the same direction as *E* one observes a decrease of *V*_*myo*_ (hyperpolarization). If ∇σ_*myo*_ points in the opposite direction of *E*, the current application leads to an increase of *V*_*myo*_ (depolarization). We extend this description for *V*_*fib*_ using a decomposition of the second right hand term of Equation (14):

(18) $$\begin{array}{r} \frac{\partial V_{fib}}{\partial t}\propto \;-\left(\nabla \sigma_{fib}\right)\cdot E+\sigma_{fib}\nabla^{2}\phi_{e} \end{array}$$

In our setup, due to the variations of the volume fraction of the myocytes and fibroblasts, ∇σ_*myo*_ is pointing in the opposite direction of *E* at point 1 of the domain, which results in a depolarization of *V*_*myo*_. On the contrary, ∇σ_*fib*_ at point 1 is aligned with E, which results in a hyperpolarization of *V*_*fib*_ at point 1.

However, this simple mechanistic explanation ignores the coupling term between the fibroblast and the myocyte domain. The current *I*_*myo, fib*_, which is determined by the parameter *R*_*myo, fib*_ and the voltage between the domains, explains why in some cases, *V*_*fib*_ follows *V*_*myo*_. In particular, at high coupling between myocyte and fibroblast domain (i.e., low values of *R*_*myo,fib*_), *V*_*myo*_ is very similar to *V*_*fib*_. Interestingly, at intermediate values of *R*_*myo,fib*_, the simulations revealed a competition of the two antagonistic effects for *V*_*fib*_ (Figures 4, 6). These two effects can even cancel each other out in a sort of tug of war situation, leaving *V*_*fib*_ at the patch boundary apparently unaffected by the application of the current (Figure 9F).

We note that the arguments presented here are valid in any spatial dimension. Based on the same arguments we also explain the mechanism behind the decrease of the virtual electrode strengths for large fibroblast-myocyte coupling. Our studies showed that due to heterogeneity of conductivity at the patch boundary, *V*_*fib*_ and *V*_*myo*_ tend to change in opposite directions. One membrane is depolarizing and the other one is hyperpolarizing, but the effect of the heterogeneities can be canceled out and even be inverted for *V*_*fib*_ because of current flow between the fibroblast and myocyte domain. This flow is lowering the effect of the conductivity heterogeneities and the virtual electrode strength. Theoretically, the current flow can go in either direction. If the fibroblast is the dominant constituent of the cardiac tissue at some heterogeneities, *V*_*fib*_ could pull *V*_*myo*_ to follow its electric depolarization or hyperpolarization. With the parameters applied in this study, myocytes were volume-wise dominant in the tissue. Thus, we did not observe such behavior.

For the 1D simulations, we reported extrema of the time evolution of Δ*V*_*fib*_ during the extracellular current application. In Figures 4B,D, the reported Δ*V*_*fib*_ is the largest absolute value (keeping its sign) of the monitored difference. These indicators exhibited a discontinuous behavior for intermediate values for the *R*_*myo,fib*_ as exemplified in Figures 4B,D. The discontinuity is caused by the transition between dominant minima and maxima of *V*_*fib*_ as illustrated in Figure S2B. The transition occurred at decreased *R*_*myo,fib*_, when increasing ${\bar{\sigma }}_{fib}$. This can be explained by the fact that the pull exerted by the myocytes on the fibroblasts diffuses faster when the diffusion is increased and is therefore less effective.

The 1D simulations for the large fibrosis revealed a complex behavior of *V*_*myo*_. In Figures 5A,C, discontinuities occurred at intermediate values of *R*_*myo, fib*_. This was caused by block of the propagating waves prior to the extracellular current application at the fibrotic patch, which modified the values of *V*_*myo*_. Though this effect is important for the dynamics of the system, in the context of our study it did affect only marginally Δ*V*_*fib*_ and Δ*V*_*myo*_.

In our 2D simulations, the influence of patch geometry on Δ*V*_*fib*_ was small (Figures 11C,D). This can be explained by the fact that the space constant for the fibroblast model is very small (computed previously around 0.15 mm) vs. the patch size of 1.5 mm. We refer the reader to Bittihn et al. (2012) for a more detailed description of the underlying mechanism.

A straightforward conclusion from our study is that fibrotic patches, similar as endo- and epicardial surfaces as well as blood vessels, constitute a potential site for wave initiation in response to defibrillation. This finding could help in parameterization of defibrillators. We suggest that further studies will lead to defibrillator protocols specifically for patients with known myocardial fibrosis to ensure defibrillation success. Also, based on our findings we propose that knowledge in the spatial distribution of fibrosis is valuable for optimization of positions for defibrillation electrodes. Potential clinical benefits of accounting for cardiac fibrosis are reduction in myocardial injury due to optimized defibrillation energy.

Prior studies on modeling of effects of fibrosis aimed at characterization of conduction and conduction defects as well as arrhythmogenesis and maintenance of arrhythmia in the atria and ventricles. Fibrosis was introduced in the models with various approaches including embedding of conduction barriers of various sizes and geometries, reduction of inter-myocyte coupling, regional replacement of myocytes with fibroblasts or myofibroblasts, and incorporation of myocyte-fibroblast coupling (Fishler, 1998; Fishler and Vepa, 1998; Mcdowell et al., 2012; Costa et al., 2014; Zeigler et al., 2016). Here, we applied the extended bidomain model to integrate various aspects of fibrotic remodeling.

A difficulty of applying the extended bidomain model is that we have only vague knowledge on parameters related to the fibroblast domain, in particular, the parameters *R*_*myo, fib*_ and σ_*fib*_. Quantitative measurements of these parameters have not been performed in normal and diseased myocardium (Kohl and Gourdie, 2014). However, a small number of *in vitro* measurements provided quantitative information to constrain these parameters. Beyond those, qualitative and indirect evidence suggests that etiologies and the progression of fibrotic remodeling affect the degree of myocyte-fibroblast and fibroblast-fibroblast electrical coupling in diseased myocardium. For these reasons, computational studies with the extended bidomain model and other computational modeling approaches commonly applied measurements from *in vitro* studies to justify parameter settings or varied parameters to reconstruct experimental findings. In this study, we chose to vary parameters within a wide range to go beyond the *in vitro* measurements and comprehensively explore potential effects of coupling.

We varied *R*_*myo, fib*_ from 1 MΩ to 1 TΩ to describe strong and negligible electrical coupling, respectively, between fibroblasts and myocytes. The lower limit is close to the junctional resistance of a myocyte pair isolated from rabbit ventricular tissue (0.81 MΩ) (Kieval et al., 1992). The upper limit led to negligible currents between the fibroblast and myocyte domain, and effectively represents the case of electrical uncoupling of the domains. Experimental measurements on myocyte-fibroblast pairs in 24 h-old culture of neonatal rat hearts cells suggest that *R*_*myo, fib*_ is variable and within a range of 125 MΩ and 3.23 GΩ (Rook et al., 1992). The variability was explained by the variable number of gap junction channels of the myocyte-fibroblast pairs. Prior modeling work applied measurements of gap junction channel conductances in the same preparation and assumed 10 to 30 gap junction channels per cell pair to vary *R*_*myo, fib*_ from 1.11 to 3.33 GΩ for simulation of fibroblast effects in the sinoatrial node (Rook et al., 1989; Kohl et al., 1994). Assuming 0 to 75 gap junctions per cell pair, *R*_*myo, fib*_ was varied from 0.44 to ∞ GΩ in a modeling study of fibroblast effects on conduction (Jacquemet and Henriquez, 2007). Based on the prior experimental and computational studies, the lower *R*_*myo, fib*_ limit (1 MΩ) in our studies appears small. We note that effects of *R*_*myo, fib*_ between 1 and 10 MΩ on virtual electrodes were in general similar. This range represents the extreme case of strong myocyte-fibroblast coupling. Increasing the lower limit of the parameter range to e.g., 1 GΩ does not change our conclusions on the relationship between *R*_*myo, fib*_ and virtual electrodes.

We varied ${\bar{\sigma }}_{fib}$ from 0 to 0.5 S/m to describe absence of electrical coupling and strong coupling, respectively, within the fibroblast domain. The lower limit represents the case of electrical uncoupling of fibroblasts. The upper limit is identical to our setting of ${\overset{\bar{}}{\sigma }}_{myo}$. Dependent on *Vol*_*fib*_ (Equation 9), this led to a range of σ_*fib*_ of 0 and up to 0.1 S/m in the region with high fibrosis with *Vol*_*fib*_ of 20%. An experimental framework for estimating σ_*fib*_ comprises measurements of junctional conductance in pairs of cultured rat cardiac cells. Conductances of fibroblast-fibroblasts pairs were in the range of 175 pS and 6 nS (Rook et al., 1992). Measurements of conductance in myocyte-myocyte pairs were up to 42 nS (Rook et al., 1990). Estimates of conductance based on gap-junctional area in myocyte pairs range between 0.9 and 216.6 nS. These measurements and estimates of conductance between cell pairs are not sufficient to determine σ_*fib*_ in normal and diseased myocardium. Further information on fibroblast geometry, gap junction distribution and cytosolic conductivity would be required to estimate σ_*fib*_ using computational approaches introduced by us previously (Bauer et al., 2013; Greiner et al., 2018). Considering that conductances of freshly isolated ventricular myocyte-myocyte pairs from control rabbit hearts were much higher (1.24 ± 0.25 μS) (Kieval et al., 1992), it is also unclear how the *in vitro* measurements relate to conductance in native myocardium. However, based on this information and the explored volume fractions, we argue that σ_*fib*_ cannot be larger than σ_*myo*_ even in the region of high fibrosis with identical volume fractions for the fibroblast and myocyte domain. In this region, our calculations (Equations 8, 9) using the maximal ${\bar{\sigma }}_{fib}$ (0.5 S/m) led to identical σ_*fib*_ and σ_*myo*_ (0.1 S/m). Prior modeling studies of electrical conduction in human atrial tissues applied σ_*fib*_ in the range of 0.02–0.1 S/m (Greisas and Zlochiver, 2016). Also, σ_*fib*_ was set to 0.06 S/m in a study on rabbit heart (Corrias et al., 2012). Based on these arguments and the prior work, we limited the exploration of σ_*fib*_ to values up to 0.1 S/m in our simulations. We note that this value is the maximum in our simulations and it is likely that smaller values in the explored range are more realistic. For small *Vol*_*fib*_ and thus sparse inter-fibroblast coupling, we point the reader at our simulations with σ_*fib*_ equal to zero.

We note that, despite the large flexibility of the extended bidomain model to describe tissue remodeling, the computational demands of the model are similar as for conventional bidomain models. A minor increase of computational demands is caused by the integration of the fibroblast domain.

We suggest that the extended bidomain model provides a more comprehensive framework for modeling of conduction in control and diseased tissue than the conventional bidomain model. While the additional parameters enable already more detailed parameterization, various refinements can be envisioned. For instance, an alternative method to define the effective volume density (1/m^3^) of electrical connections between fibroblasts and myocytes is:

(19) $$\begin{array}{r} \beta_{myo,fib}=\beta^{\left(r\right)\;}\frac{\;Vol_{fib}\;}{Vol_{fib,single}\;} \end{array}$$

Because the electrical coupling between the fibroblasts and myocytes is mainly unknown and potentially varies from one situation to another we can define a parameter, β^(*r*)^ (dimensionless), for characterizing this coupling. A value of β^(*r*)^ = 1 corresponds to a situation where each of the fibroblasts has an electrical connection with the myocyte domain. A larger (smaller) value of β^(*r*)^ allows changing this situation. For β^(*r*)^ < 1 not all the fibroblasts are coupled to a myocyte and conversely for β^(*r*)^ > 1 each fibroblast is coupled to more than one myocyte. Both, *R*_*myo, fib*_ and β^(*r*)^ are factors that contribute to the coupling between myocytes and fibroblasts and hence their effective influence can be combined in a single variable, β^(fract)^ defined as:

(20) $$\begin{array}{r} \beta^{\left(fract\right)}\;=\;\frac{\beta^{\left(r\right)\;}}{R_{myo,fib}} \end{array}$$

### Limitations

We acknowledge several limitations related to the presented work. The studies were performed in 1D and 2D domains with isotropic conductivities. We did not consider unequal anisotropy ratios of conductivities. We applied simple descriptions of fibrotic remodeling derived from a rabbit model of cardiac infarction. While fibrotic remodeling is known to be a multi-faceted process comprising e.g., activation of fibroblasts, their differentiation into myofibroblasts, and subcellular remodeling of structural und electrophysiological properties, we primarily considered increased volume ratios of cells and the extracellular space in our study. Also, we applied mathematical models of normal myocytes and fibroblasts, and did not adjust these models to account for fibrosis and associated remodeling.

A limitation of our work is related to incomplete understanding of myocyte-fibroblast and fibroblast-fibroblast coupling in the heart. As discussed above, experimental measurements exist only for *in vitro* models and it is difficult to apply these measurements for parameterization of models of fibrotic remodeling in native myocardium. Also, we applied a simple linear model for scaling of σ_*fib*_ by *Vol*_*fib*_ (Equation 9). Non-linear models might be more realistic. In particular, for small *Vol*_*fib*_, it is likely that fibroblasts are not electrically coupled with other fibroblasts, which can be reflected by a model using piece-wise defined functions. We note the controversy on myocyte-fibroblast coupling and the contribution of fibroblasts in physiological and pathophysiological conduction (Kohl and Gourdie, 2014). Based on our simulations, myocyte-fibroblast coupling is a major determinant of virtual electrode strength. However, for the entire simulated range of myocyte-fibroblast coupling, boundaries of fibrotic patches were associated with virtual electrodes.

In our comparison of software for extended bidomain modeling, we confirmed that the new and our prior implementation yielded very similar results when simulating a homogenous tissue. Peak *V*_*myo*_ and *V*_*fib*_ differed only by 5.08% (13.98 vs. 13.3 mV) and 5.23% (13.69 vs. 13.03 mV), respectively. However, we noticed that the approach for discretization of the patch boundary affects the magnitude of the altered membrane voltages. Conventional finite difference discretization (Sachse, 2004) led to significant differences vs. the finite volume discretization used in our studies. We reduced differences to the finite volume method by implementation of a finite difference approximation based on Kirchhoff's law (Witwer et al., 1972). However, the appropriate modeling approach should be dictated by the spatial distribution of the structural remodeling. While our finite volume approach described structural remodeling as an abrupt change from control tissue to the fibrotic patch, a gradual approach might be more appropriate (Seidel et al., 2017).

A related limitation of our study is the geometry and size of patches explored in the 2D simulations. We focused on disk and square shaped patches with a single size. Larger effects of geometry are expected for smaller sizes, i.e., below the millimeter scale, but we leave this for a future exploration. Also, we suggest that microscopic imaging can provide insights into the detailed distribution of remodeling.

## Data Availability

The datasets generated for this study are available on request to the corresponding author.

## Author Contributions

JB and FS designed the study. All authors developed software for this project, analyzed, and interpreted the data, drafted and critically revised the manuscript, and approved the version to be published.

### Conflict of Interest Statement

The authors declare that the research was conducted in the absence of any commercial or financial relationships that could be construed as a potential conflict of interest.
