# Supplementary material for: Extended Bidomain Modeling of Defibrillation: Quantifying Virtual Electrode Strengths in Fibrotic Myocardium
Source: Front Physiol. 2019 Apr 3;10:337. doi: 10.3389/fphys.2019.00337 (PMC6456788; doi:10.3389/fphys.2019.00337)
Supplement: Supplementary file 1 [file Data_Sheet_1.pdf]

Supplemental Tables

Table S1: Effects of dx on simulation of conduction and extracellular current application. The 1D setup was used. $R_{myo,fib}$ and $\bar{\sigma}_{fib}$ were set to 10^6 MΩ and 0.1 S/m, respectively. The values given in parentheses indicate a 95% confidence interval for the measurements. Conduction velocity was measured for the final wave, prior to the extracellular current application. The space constants were measured by fitting.

dx (µm)	100	50	25
**Conduction velocity (cm/s)**	(54.05 - 54.34)	(55.15 - 55.35)	(55.48 - 55.63)
$\Delta V_{myo}^{(1)}$ (mV)	+9.356	+9.363	+9.365
$\Delta V_{myo}^{(2)}$ (mV)	-8.365	-8.398	-8.403
$\Delta V_{fib}^{(1)}$ (mV)	-4.197	-4.262	-4.277
$\Delta V_{fib}^{(2)}$ (mV)	+4.129	+4.192	+4.207
$E_{applied}$ (V/cm)	0.547	0.547	0.548
λ_{myo} (mm)	(0.617 - 0.664)	(0.597 - 0.627)	(0.600 - 0.620)
λ_{fib} (mm)	(0.175 - 0.185)	(0.177 - 0.184)	(0.173 - 0.180)

Table S2: Effects of dx on simulation of conduction and extracellular current application. The 1D setup was used. $R_{myo,fib}$ and $\bar{\sigma}_{fib}$ were set to 10^3 MΩ and 0.1 S/m, respectively.

dx (µm)	100	50	25
**Conduction velocity (cm/s)**	(51.79 – 52.88)	(52.95 – 53.75)	(53.75 - 53.90)
$\Delta V_{myo}^{(1)}$ (mV)	+8.370	+8.407	+8.421
$\Delta V_{myo}^{(2)}$ (mV)	-7.483	-7.527	-7.542
$\Delta V_{fib}^{(1)}$ (mV)	-0.869	-1.089	-1.143
$\Delta V_{fib}^{(2)}$ (mV)	+0.867	+1.086	+1.140
$E_{applied}$ (V/cm)	0.546	0.547	0.547
λ_{myo} (mm)	(0.560 - 0.570)	(0.569 - 0.574)	(0.562 - 0.579)
λ_{fib} (mm)	(0.073-0.073)	(0.060 - 0.103)	(0.071 - 0.087)

Table S3: Effects of dx on simulation of conduction and extracellular current application. The 1D setup was used. $R_{myo,fib}$ and $\overline{\sigma}_{fib}$ were set to 1 MΩ and 0.1 S/m, respectively.

dx (µm)	100	50	25
**Conduction velocity (cm/s)**	(41.81 - 42.51)	(42.995 – 43.515)	(43.37 - 43.65)
$\Delta V_{myo}^{(1)}$ **(mV)**	+7.777	+7.804	+7.818
$\Delta V_{myo}^{(2)}$ **(mV)**	-7.162	-7.194	-7.208
$\Delta V_{fib}^{(1)}$ **(mV)**	+7.755	+7.763	+7.741
$\Delta V_{fib}^{(2)}$ **(mV)**	-7.141	-7.153	-7.132
$E_{applied}$ **(V/cm)**	0.546	0.546	0.546
λ_{myo} **(mm)**	(0.514 - 0.537)	(0.531 - 0.544)	(0.529 - 0.538)
λ_{fib} **(mm)**	(0.515 - 0.539)	(0.532 - 0.547)	(0.530 - 0.541)

Table S4: Results from 2D simulations for parameters $\overline{\sigma}_{fib}$=0.1 S/m and dx=50 µm.

	$R_{myo,fib}$ **(MΩ)**	**1**	**10**	**10^2**	**10^3**	**10^4**	**10^5**	**10^6**
**Square**	$\Delta V_{myo}^{(1)}$**(mV)**	7.435	7.447	7.491	7.912	8.652	8.804	8.791
	$\Delta V_{myo}^{(2)}$ **(mV)**	-6.957	-6.971	-6.999	-7.304	-8.034	-8.108	-8.052
	$\Delta V_{fib}^{(1)}$ **(mV)**	7.395	7.106	5.583	0.452	-4.163	-4.716	-4.307
	$\Delta V_{fib}^{(2)}$ **(mV)**	-6.905	-6.539	-4.867	0.123	3.289	4.040	4.571
	E **(V/cm)**	0.530	0.530	0.530	0.530	0.530	0.530	0.530
**Disk**	$\Delta V_{myo}^{(1)}$ **(mV)**	7.178	7.189	7.224	7.589	8.254	8.396	8.384
	$\Delta V_{myo}^{(2)}$ **(mV)**	-6.747	-6.758	-6.791	-7.057	-7.737	-7.816	-7.756
	$\Delta V_{fib}^{(1)}$ **(mV)**	7.140	6.854	5.359	0.270	-4.315	-4.862	-4.454
	$\Delta V_{fib}^{(2)}$ **(mV)**	-6.696	-6.333	-4.696	0.280	3.446	4.198	4.728
	E **(V/cm)**	0.530	0.530	0.530	0.530	0.530	0.530	0.530

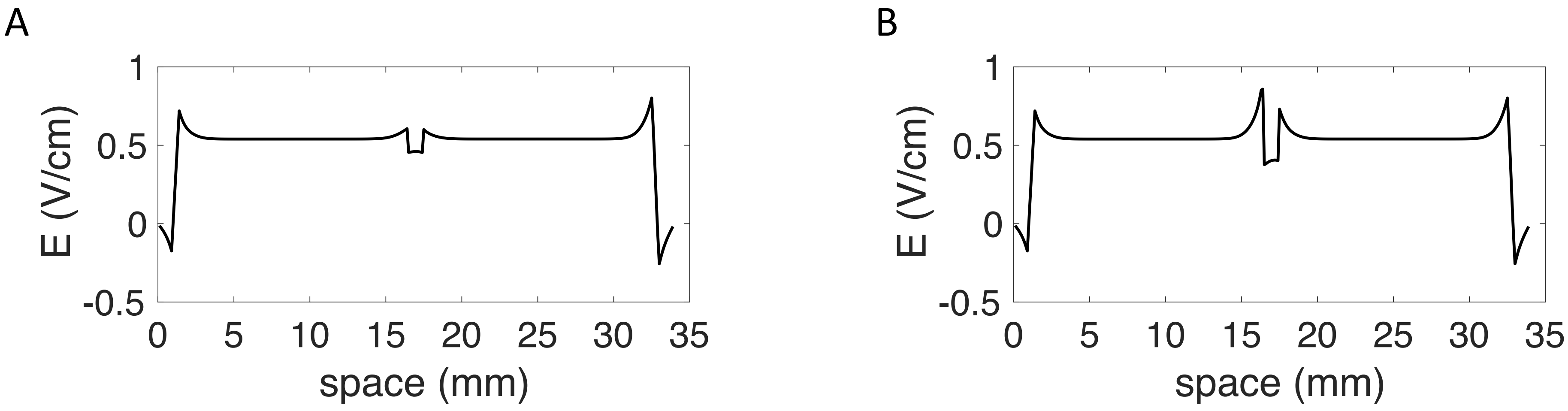

Figure S1: Spatial plot of E at end of current application in 1D model with (A) average and (B) large fibrosis.

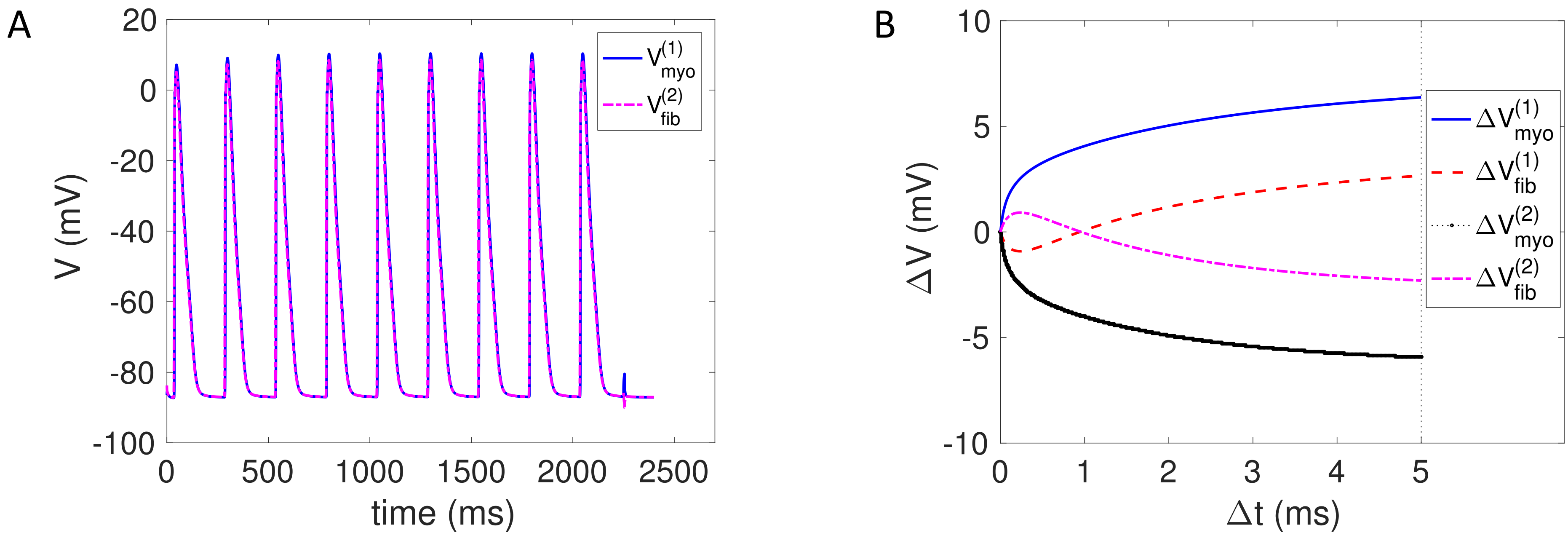

Figure S2: Simulation with $R_{myo,fib}$=100 MΩ and $\bar{\sigma}_{fib}$=0.5 S/m. (A) Time courses of $\Delta V^{(1)}_{myo}$ and $\Delta V^{(2)}_{fib}$. (B) Time course of ΔV at point 1 and 2 for both myocytes and fibroblasts. E during the current application was 0.536 V/cm. Δt measures the time during the current application (5 ms duration). The vertical line at Δt=5 ms indicates the end of the current application. Note that the ΔV_{fib} are not monotonous functions of time for intermediate values of $R_{myo,fib}$.

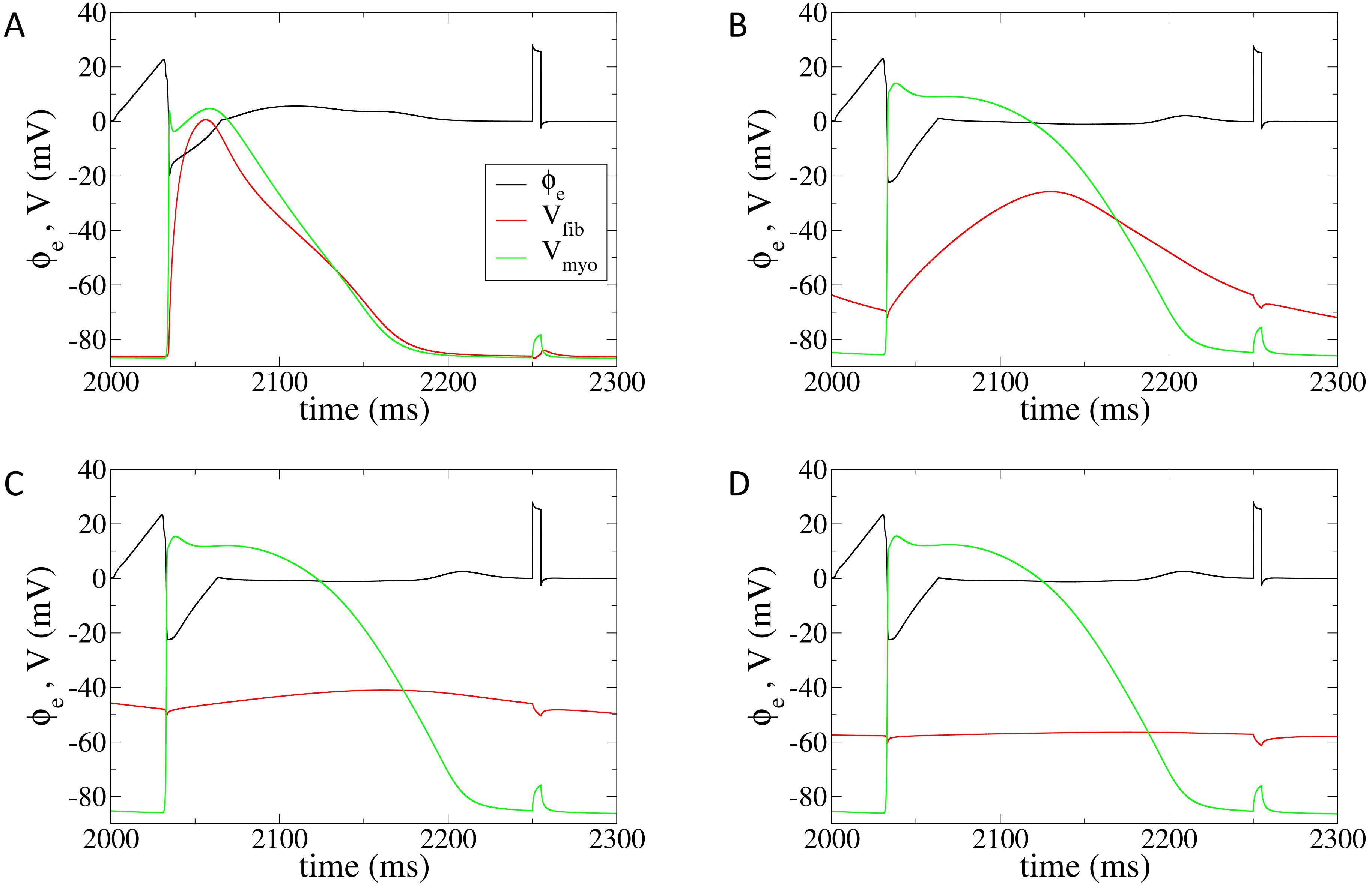

Figure S3: Simulated ϕ_e , V_{myo} and V_{fib} in response to intracellular and extracellular current application at left boundary of fibrotic patch (x=16.5 mm). Parameters were set for the average fibrotic case with $\overline{\sigma}_{fib}$=0.1 S/m. $R_{myo,fib}$ was set to (A) 1 GΩ, (B) 22 GΩ, (C) 150 GΩ, and (D) 1 TΩ. The simulation in (B) yielded the maximal value of V_{myo} in Fig. 3A. The simulation in (C) yielded the maximal value of V_{fib} in Fig. 4A.
